# The prognostic value of long non coding RNAs in non small cell lung cancer: A meta-analysis

**DOI:** 10.18632/oncotarget.13223

**Published:** 2016-11-08

**Authors:** Manni Wang, Xuelei Ma, Chenjing Zhu, Linghong Guo, Qingfang Li, Ming Liu, Jing Zhang

**Affiliations:** ^1^ State Key Laboratory of Biotherapy and Cancer Center, West China Hospital, Sichuan University, Chengdu, PR China; ^2^ West China Hospital, Sichuan University, Chengdu, PR China

**Keywords:** lncRNAs, NSCLC, prognosis, meta-analysis

## Abstract

**Background:**

Reports have demonstrated the prognostic function of long non-coding RNAS (lncRNAS) in patients with cancer. However, their prognostic functions in non small cell lung cancer (NSCLC) remain controversial. We therefore performed a meta-analysis on six lncRNAs (PVT1, AFAP1-AS1, LINC01133, ANRIL, MEG3 and UCA1) to clarify their prognostic roles in NSCLC.

**Results:**

Thirty-six studies involving 6267 patients with NSCLC and 34 lncRNAs were included. Of the listed lncRNAs, 20 were shown to negatively affect patients' overall survival while the high expression of 13 lncRNAs indicated better survival outcomes.

**Materials and Methods:**

The log-rank *p value* and Kaplan–Meier survival curves of survival outcomes were extracted for hazard ratio (HR) calculation. Survival outcomes were measured by overall survival (OS) and event free survival (EFS) which were then analyzed by calculating pooled hazard ratios. The heterogeneity was detected by Q statistic and I-squared statistic.

**Conclusions:**

The abnormal expression of lncRNAs may significantly affect NSCLC patients' survival and may serve as a novel predictive factor for prognosis of NSCLC patients.

## INTRODUCTION

Lung cancer is one of the most common causes of cancer-related deaths worldwide and non small cell lung cancer (NSCLC) accounts for 80% of all cases [1]. GLOBOCAN 2012 reported that there were approximately 14.1 million cancer patients in the world and 8.2 million of them died in 2012, most of which were population from less developed countries [2]. Patients with lung cancer are usually diagnosed at advanced stages with relatively poor prognosis. The estimated overall 5-year survival rate of advanced stage lung cancer is 0–14% [3, 4], while the 5-year survival rate of early stage NSCLC can be as high as 83%., which informs us that the early diagnosis and the finding of new molecular targets for NSCLC are the key to improve clinical strategies and outcomes of NSCLC [5]. Long non-coding RNAs (lncRNAs) are non-protein-coding RNA molecules with a length of more than 200 nucleotides and often expressed in a spatial, temporal and tissue-specific pattern [6, 7]. In the past, lncRNAs were merely viewed as transcriptional “noise” [8]. Recently, a growing number of genome-wide transcriptome studies have identified about 3000 lncRNASs and at the same time indicated their diverse biological functions in both normal and degenerated tissues, including cell growth, differentiation and disease progression [9]. lncRNAs may act as primary regulators of the molecular interaction with DNA-binding proteins and epigenetically regulate the expression of target genes [10].

So far, controversy about the prognostic role of lncRNAs in NSCLC still exists. Some studies drew statistically insignificant conclusions [11, 12], while some studies showed that lncRNAs could be important biomarkers for the assessment of overall survival and recurrence. Due to the limitation of sample size and research number, a single study may not be able to reflect the facts accurately. Therefore, we conducted a meta-analysis to identify the exact role of lncRNAs in NSCLC patients' prognosis. At the same time, we summarized in our study the relation of different lncRNAs to patients' prognosis. Kaplan–Meier survival analysis and log-rank tests were performed in our enrolled studies to further evaluate the correlation between lncRNA expression and the prognosis of NSCLC patients. Pooled results indicated that lncRNAs played an important role in NSCLC overall survival time, which provided us with new insights in the therapeutic strategies of NSCLC.

## RESULTS

### Study selection

**Figure 1:**
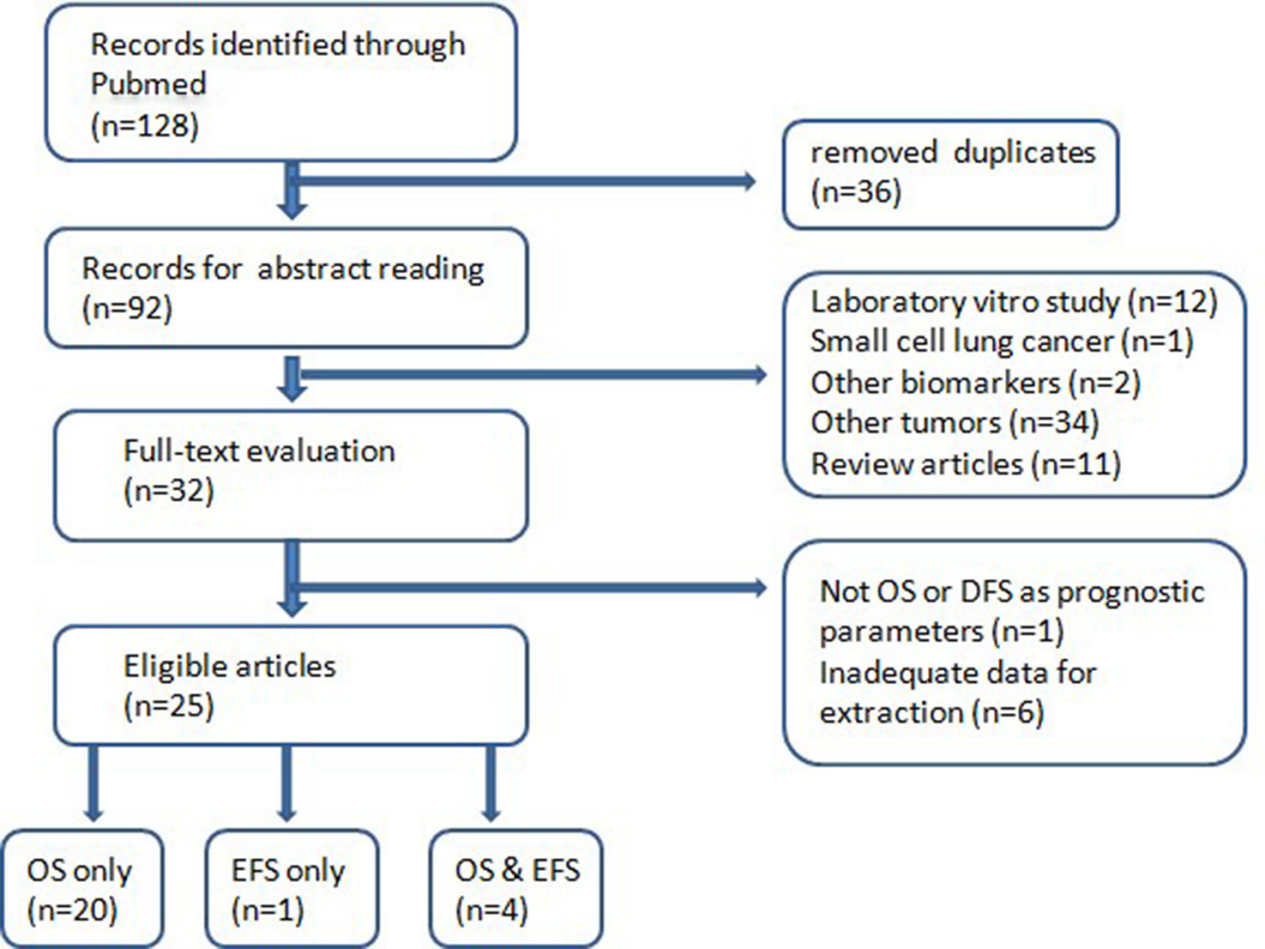
The flow chart of selection process

After full-text assessment of all included articles, we excluded studies that did not use EFS or OS as survival parameters. Studies that lacked information for calculation with methods developed by Parmar, Williamson , and Tierney (Parmar et al., 1998; Williamson et al., 2002; Tierney) were also excluded. The initial search returned 128 articles, from which 36 duplicated records were removed. Abstracts of the remaining 92 articles were carefully read by two authors independently and we excluded 60 unqualified literatures: laboratory studies (*n* = 12), review articles (*n* = 11), other biomarkers (*n* = 2) and other types of cancer (*n* = 35). Next we went through the full texts of the remaining 32 studies and 25 with adequate data for calculation were finally enrolled. The flow chart of selection process is shown in Figure 1. The supplementary search returned 46 articles, 11 of which contain useful information.

### Characteristics of included studies

Among the 36 studies, one article [11] used progression free survival instead of disease free survival, we therefore combined DFS and PFS together and use event free survival EFS as prognosis parameter of our study. 28 studies used overall survival OS as prognosis parameter, one study used event free survival EFS and four contained OS as well as EFS. All 36 studies used the quantitative real-time reverse transcription-PCR (qRTPCR) method to measure the expression of lncRNAs in tumor samples. All the included studies analyzed the prognosis of 6267 patients with NSCLC and the correlation between 34 lncRNAs levels and survival outcomes. All necessary data of included trials are listed in Table 1 and Table 2.

**Table 1:** Criteria for the inclusion of prognostic lncRNA studies

Study design	Prospective or retrospective cohort
Time of study	After 2006
Tumor type	Non-small cell lung cancer (NSCLC)
Assay type	Tumor tissue or blood sample
RNA measurement	qRT-PCR or ISH
Outcome type	Overall Survival (OS) or Disease Free Survival (DFS)
Included results	Univariate and multivariate survival analysis (Cox proportional hazards regression model) including HRs, 95% CIs and *P* value / Kaplan Meier survival curves with enough data for calculation
Population size	≥ 30
Length of follow-up	≥ 1 year
Source	Peer-reviewed journals
Language	English

Abbreviations: OS, overall survival; DFS, disease-free survival; CI, confidence interval.

**Table 2:** Frequency of studies assessing prognostic value of lncRNAs in NSCLC

Name of lncRNA	Number of studies	Reference
PVT1	2	Yan-Rong Yang,
		Di Cui
CASC2	1	Xuezhi He
PANDAR	1	L han
SPRY4-IT1	1	Sun M
TUG1	1	Eb Zhang
AFAP1-AS1	2	Jun Deng
		Zhaoyang Zeng
ANRIL	2	Ling Lin
		Feng-qi Nie
BANCR	1	Ming Sun
CARLo-5	1	Jie Luo
GAS6-AS1	1	Liang Han
H19	1	Erbao Zhang
HMlincRNA717	1	Xiao Xie
HOTAIR	2	Xiang-hua Liu
		Takayuki Nakagawa
LINC01133	2	Jing Zhang
		Chongshuang Zang
MALAT-1	2	Lars Henning Schmidt
		Liqin Shen
Sox2ot	1	Zhibo Hou
UCA1	3	Huimin Wang
		Ningning Cheng
		Wei Nie
MEG3	2	Lin Su
		Kaihua Lu
BC087858	1	Hui Pan
XIST	1	Jing Fang
NEAT1	1	Chengcao Sun
HNF1A-AS1	1	Ying Wu
MVIH	1	Feng-qi Nie
LINC00342	1	Li Wang
LINC00473	1	Zirong Chen
TUSC7	1	Zhongwei Wang

The number of patients enrolled in each study ranged from 20 to 1926, and the follow-up duration varied from 25 months to 200 months. Among them, 33 studies involved participants from China [11–43] and three studies involved patients respectively from Japan [44], Germany [45] and USA [46]. All studies investigated patients with NSCLC and qRT–PCR was used to detect lncRNAs expression in tumor tissues.

### Overall Analyses

20 lncRNAs were shown to negatively affect patients' overall survival while 13 lncRNAs were associated with better survival outcomes. One study [11] on ANRIL (Nie et al: OS HR = 2.23 , 95% CI: 0.89–5.59, *P* = 0.09) showed no significant prognostic effect of lncRNAs expression on patients' overall survival. Wang et al. [12] observed no correlation between the expression of TUSC7 and patients' DFS, but significant correlation between TUSC7 expression and patients' OS. The BC087858 expression level was also associated with prognosis but it just reached the marginal statistical significance (*P* =0.083) [38]. All HRs, 95% CI and *P* values of included studies are listed in Table 3.

**Table 3:** Basic information of included studies

Author	LncRNA	Population	Sample	Total patients	Assay	Survival analysis	Follow up(month)
Takayuki N	HOTAIR	Japan	Tumor tissue	77	qRT-PCR	EFS	40–50
Xianghua Liu	HOTAIR	China	Tumor tissue	42	qRT-PCR	OS	60
YanRong Yang	PVT1	China	Tumor tissue	82	qRT-PCR	OS	60
Di Cui	PVT1	China	Tumor tissue	108	qRT-PCR	OS,EFS	30–40
Jun Deng	AFAP1-AS1	China	Tumor tissue	121	qRT-PCR	OS	60
Zhaoyang Zeng	AFAP1-AS1	China	Tumor tissue	332	qRT-PCR	OS	96–168
Jing Zhang	LINC01133	China	Tumor tissue	39	qRT-PCR	OS	60
Chongshuang Zang	LINC01133	China	Tumor tissue	68	qRT-PCR	OS	30–40
Ling Lin	ANRIL	China	Tumor tissue	87	qRT-PCR	OS	60
Fengqi Nie	ANRIL	China	Tumor tissue	68	qRT-PCR	OS,EFS	36
Xuezhi He	CASC2	China	Tumor tissue	76	qRT-PCR	OS	60
L han	PANDAR	China	Tumor tissue	140	qRT-PCR	OS	60
Sun M	SPRY4-IT1	China	Tumor tissue	121	qRT-PCR	OS,EFS	30–40
Eb Zhang	TUG1	China	Tumor tissue	192	qRT-PCR	OS	60
Ming Sun1	BANCR	China	Tumor tissue	113	qRT-PCR	OS	36
Jie Luo	CARLo-5	China	Tumor tissue	62	qRT-PCR	OS	60
Liang Han	GAS6-AS1	China	Tumor tissue	50	qRT-PCR	OS	60
Erbao Zhang	H19	China	Tumor tissue	70	qRT-PCR	OS	60
Zirong Chen	LINC00473	USA	Tumor tissue	469	qRT-PCR	OS	> 50
Zhongwei Wang	TUSC7	China	Tumor tissue	112	qRT-PCR	OS,EFS	> 60
Xiao Xie	HMlincRNA717	China	Tumor tissue	118	qRT-PCR	OS	80
Liqin Shen	MALAT-1	China	Tumor tissue	78	qRT-PCR	EFS	60
Zhibo Hou	Sox2ot	China	Tumor tissue	47	qRT-PCR	OS	60
Wei Nie	UCA1	China	Tumor tissue	112	qRT-PCR	OS	80
Fengqi Nie	MVIH	China	Tumor tissue	42	qRT-PCR	OS	36
Lars H Schmidt	MALAT-1	Germany	Tumor tissue	102	qRT-PCR	OS	100–140
Huimin Wang	UCA1	China	Tumor tissue	60	qRT-PCR	OS	60–80
Ningning Cheng	UCA1	China	Tumor tissue	52	qRT-PCR	EFS	20–25
Lin Su	MEG3	China	Tumor tissue	20	qRT-PCR	OS	60
Kaihua Lu	MEG3	China	Tumor tissue	42	qRT-PCR	OS	40–60
Hui Pan	BC087858	China	Tumor tissue	38	qRT-PCR	EFS	30
Jing Fang	XIST	China	Tumor tissue	53	qRT-PCR	OS	150–200
Chengcao Sun	NEAT1	China	Tumor tissue	96	qRT-PCR	OS	40
Ying Wu	HNF1A-AS1	China	Tumor tissue	856	qRT-PCR	OS	200
Li Wang	LINC00342	China	Tumor tissue	1926	qRT-PCR	OS	200
Meng Zhou	CTD-2358C21.4	China	Tumor tissue	196	qRT-PCR	OS	60
	RP11-94L15.2						
	KCNK15-AS1						
	AC104134.2						
	RP11-21L23.2						
	GPR158-AS1						
	RP11-701P16.5						
	RP11-379F4.4						

After careful reading of 92 literatures of the first search after duplicates were removed and all literatures returned from the second search, we summarized all lncRNAs up to date whose prognostic roles in NSCLC were investigated (Table 4). Of the 34 lncRNAs presented, eight( RP11-21L23.2, GPR158-AS1, RP11-701P16.5, RP11-379F4.4, CTD-2358C21.4, RP11-94L15.2, KCNK15-AS1 and AC104134.2) lacked information for calculation but their influences on prognosis were clearly demonstrated in the study [41].

**Table 4:** Summary of hazard ratios of lncRNA expression in NSCLC

LncRNA	Hazard ratio		CI		*p* value	Log (HR)	SE	Expression related to bad prognosis
	OS	EFS	Lower	Upper				
HOTAIR		3.10	1.05	9.10	0.04	1.13	0.55	High
HOTAIR	2.69		1.30	5.56	0.007	0.99	0.37	High
PVT1	3.25		1.84	5.75	< 0.0001	1.18	0.29	High
PVT1	1.72		1.01	2.91	0.05	0.54	0.27	High
		1.97	1.01	3.84	0.05	0.68	0.34	High
AFAP1-AS1	8.94		3.10	25.75	< 0.0001	2.19	0.54	High
AFAP1-AS1	1.90		1.17	3.08	0.009	0.64	0.25	High
	2.90		1.54	5.47	0.001	1.06	0.32	High
LINC01133	2.39		1.03	5.54	0.04	0.87	0.43	High
LINC01133	2.25		1.25	4.05	0.007	0.81	0.30	High
ANRIL	2.53		1.28	5.03	0.008	0.93	0.35	High
ANRIL	2.23		0.89	5.59	0.09	0.80	0.47	–
		3.53	1.64	7.57	0.001	1.26	0.39	High
H19	1.08		1.04	1.13	< 0.0001	0.08	0.02	High
MALAT-1	1.79		1.09	2.92	0.02	0.58	0.25	High
MALAT-1		2.36	1.19	4.69	0.01	0.86	0.35	High
Sox2ot	2.80		1.14	6.90	0.03	1.03	0.46	High
UCA1	1.94		1.06	3.26	0.029	0.66	0.29	High
UCA1		3.25	1.17	9.02	0.02	1.18	0.52	High
UCA1	1.40		1.07	1.85	0.02	0.34	0.14	High
MVIH	2.01		1.08	3.77	0.03	0.70	0.32	High
CARLo-5	2.20		1.20	4.05	0.01	0.79	0.31	High
LINC00473	1.73		1.27	2.37	0.0006	0.55	0.16	High
XIST	6.3		4.09	9.69	< 0.0001	1.84	0.22	High
NEAT1	1.82		1.07	3.09	0.03	0.6	0.27	High
HNF1A-AS1	1.19		1.01	1.39	0.03	0.17	0.08	High
LINC00342	1.16		1.05	1.28	0.03	0.15	0.05	High
BC087858		2.51	0.89	7.10	0.083	−	−	−
TUSC7	0.26		0.10	0.66	0.005	−1.35	0.48	Low
		0.7	0.42	1.16	0.17	−0.36	0.26	−
HMlincRNA717	0.40		0.21	0.75	0.004	−0.91	0.32	Low
CASC2	0.28		0.10	0.76	0.01	−1.29	0.52	Low
PANDAR	0.65		0.46	0.93	0.02	−0.43	0.18	Low
SPRY4-IT1	0.45		0.24	0.82	0.01	−0.80	0.31	Low
		0.44	0.26	0.73	0.001	−0.83	0.26	Low
TUG1	0.78		0.69	0.88	< 0.0001	−0.25	0.06	Low
BANCR	0.50		0.26	0.95	0.03	−0.70	0.33	Low
GAS6-AS1	0.15		0.03	0.87	0.03	−1.90	0.90	Low
MEG3	0.33		0.12	0.88	0.03	−1.11	0.5	Low
MEG3	0.26		0.12	0.57	0.007	−1.35	0.40	Low

### Subgroup analysis

Among the 20 listed lncRNASs, eight (HOTAIR, PVT1, AFAP1-AS1, LINC01133 and ANRIL, UCA1, MALAT-1, MEG3) have been studied by two or more articles. We then carried out meta analyses and obtained the combined HRs. While other studies have sufficient information for pooled analysis, studies on HOTAIR and MALAT-1 looked into OS and EFS separately and we were therefore unable to conduct relevant meta analysis.

### PVT1

**Figure 2:**
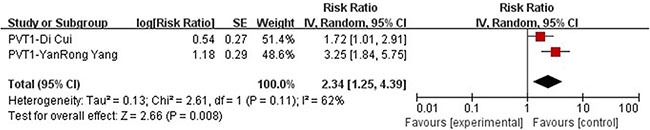
Forrest plots of studies evaluating hazard ratios of high PVT1 expression as compared to low expression

We performed meta-analysis on articles choosing lncRNA PVT1 as a prognostic marker. The two studies included in meta-analysis [14, 15], both conducted multivariate Cox regression analysis and the data such as HR is therefore directly extracted and put into pooled analysis. The median follow-up period is 41 months [14] and 32 months [15] respectively and the information of a total number of 190 patients were collected. There was evidence of considerable heterogeneity in these two groups (*P* = 0.11, I2 = 62%) so the random effect model was selected. A combined HR of 2.34 (95% CI: 1.25–4.39, *P* = 0.008) for those patients with high expression of PVT1 was found, from which we drew a conclusion that high expression of long non-coding RNAS PVT1 is a predictor of poorer overall survival (Figure 2).

### AFAP1-AS1

**Figure 3:**
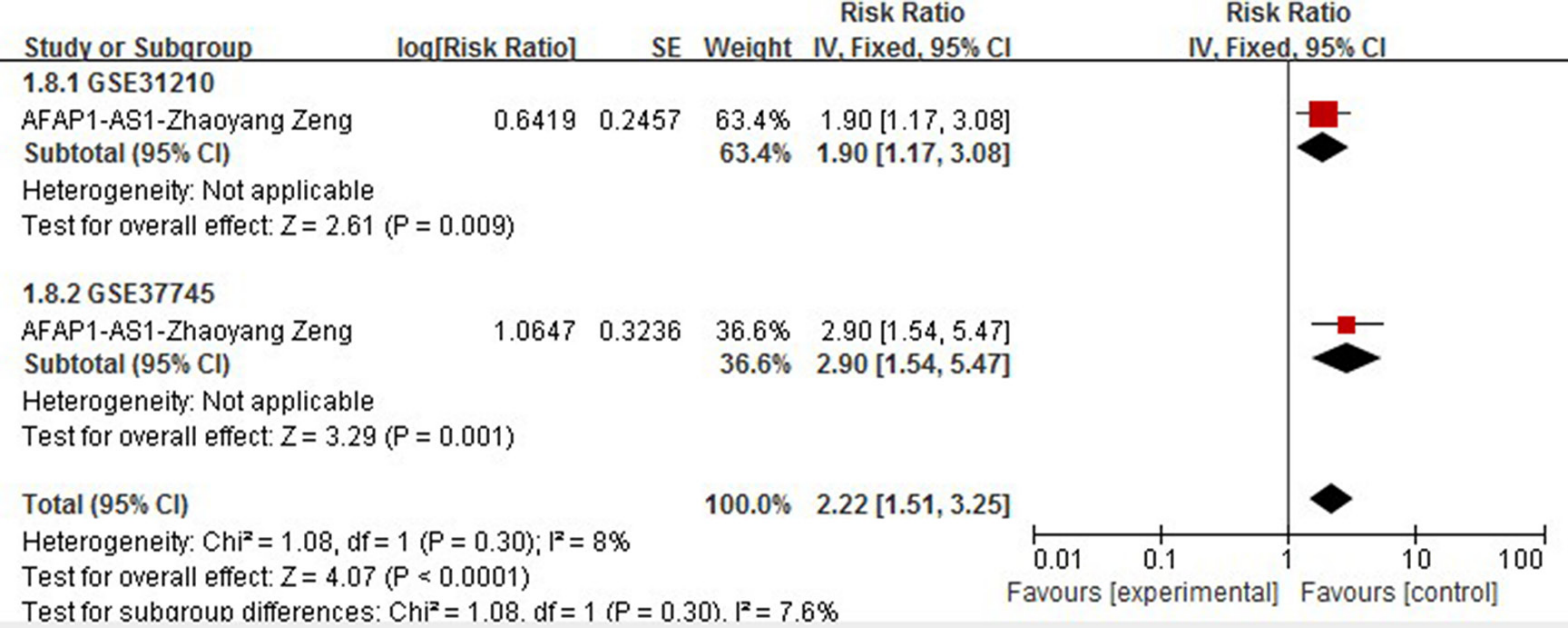
Forrest plots of studies evaluating hazard ratios of high AFAP1-AS1 expression as compared to low expression with 2 cohorts of one study

Two studies [16, 17] described the elevated expression of long non-coding RNAS AFAP1-AS1 as predictive of poor OS in NSCLC (*n* = 332). Deng et al enrolled 121 patients diagnosed with NSCLC who had never received any therapy before surgery. Multivariate Cox regression analysis was performed and HR for high AFAP1-AS1 expression was 8.947 (95% CI = 3.115–25.694, *P* = 0.000). Zang et al included two independent cohorts, GSE31210 (*N* = 226) [47] and GSE37745 (*N* = 106) [48] which had complete follow-up data. This article presented Kaplan-Meier curve with precise number of patients and the death ratio in high and low expression group, instead of Cox regression analysis result. The combined HR (HR = 2.22,95% CI: 1.51–3.25, *P* < 0.0001) from subsequent pooled analysis of these two cohorts is shown in Figure 3.

**Figure 4:**
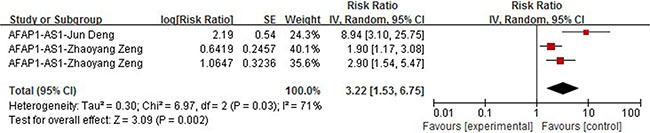
Forrest plots of studies evaluating hazard ratios of high AFAP1-AS1 expression as compared to low expression

We then carried out meta analysis with these two articles containing three groups of data and the subsequent combined HR is shown in Figure 4. Significant heterogeneity among selected studies according to *Q*-test (chi2 = 6.97) and I-squared result (I2 = 71%, *P* = 0.03) was observed, so the random model was applied to calculate a pooled HR (HR = 3.22,95% CI: 1.53–6.75, *P* =0.002), which indicated that an elevated expression level of AFAP1-AS1 was a strong predictor of poorer OS.

### LINC01133

**Figure 5:**
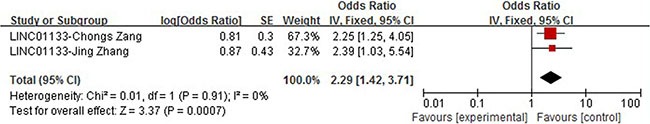
Forrest plots of studies evaluating hazard ratios of high LINC01133 expression as compared to low expression

We included two studies investigating the correlation of LINC01133 expression with patients overall survival. Ling et al studied a cohort of 79 pairs of NSCLC tumor tissues, including 39 lung squamous cell cancer (LSCC) and 40 lung adenocarcinoma (LAD). Both studies conducted Kaplan-Meier survival analysis and no significant heterogeneity was observed (I2 = 0%, *P* = 0.91). Further meta analysis using the fixed effect model revealed that high expression of LINC01133 could develop as an independent factor for predicting the prognosis of NSCLC patients (HR = 2.29, 95% CI: 1.42–3.71, *P* = 0.0007) (Figure 5).

### ANRIL

**Figure 6:**
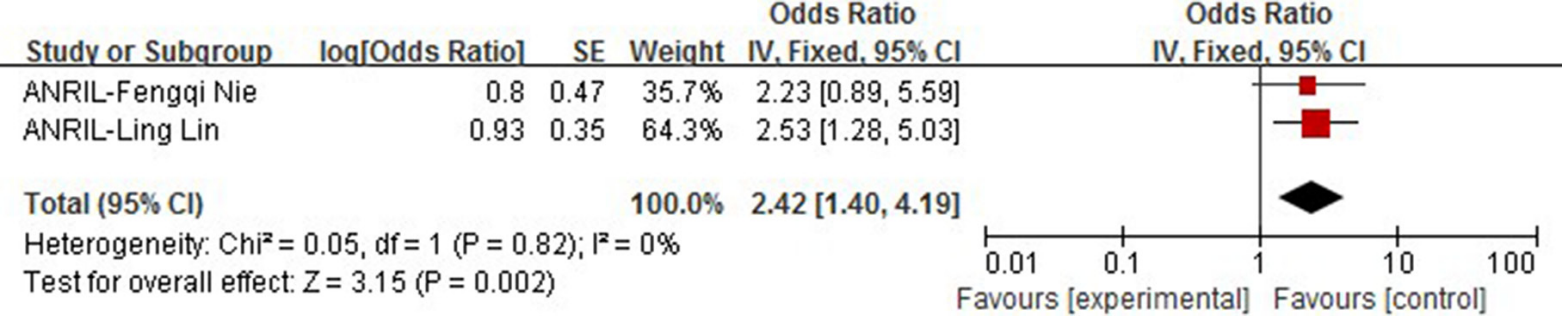
Forrest plots of studies evaluating hazard ratios of high ANRIL expression as compared to low expression

Two studies involved the multivariate Cox regression analysis of prognostic parameters including the expression of ANRIL in NSCLC patients. Ling et al (*N* = 87) and Nie et al (*N* = 68) had clinical follow-ups of 60 months and 36 months respectively. In Nie's study, the ANRIL over-expression did not show a significant influence on OS (HR = 2.23, 95% CI: 0.89–5.59, *P* = 0.09). In order to clarify the impact of ANRIL expression on patients' survival, we performed a pooled analysis. We observed no heterogeneity between studies (I2 = o%, *P* = 0.82) and therefore fixed effect model was applied to calculate the association between high tumoral ANRIL expression and OS (HR 2.42, 95% CI: 1.40–4.19, *P* = 0.002). These results suggest that high expression of ANRIL could predict worse prognosis of NSCLC patients regarding overall survival and may be an independent prognostic marker (Figure 6).

### UCA1

**Figure 7:**
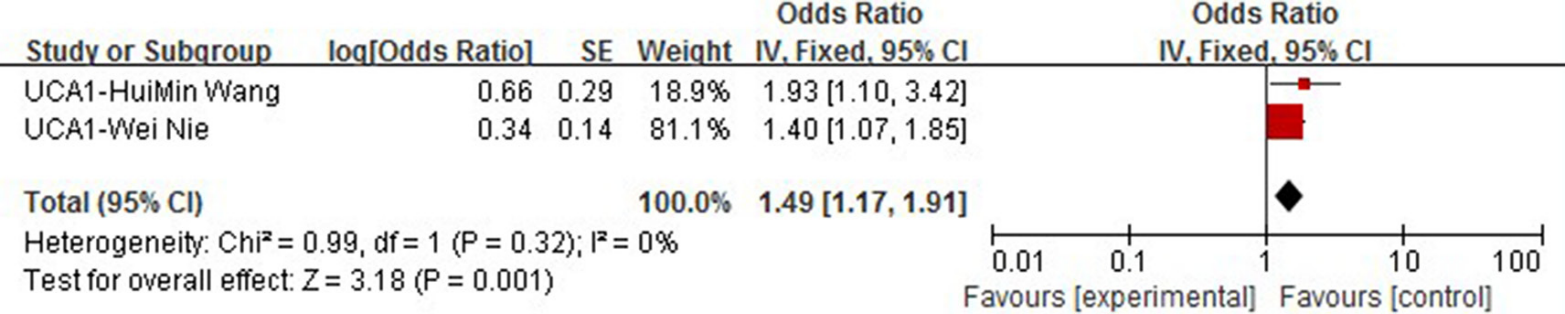
Forrest plots of studies evaluating hazard ratios of high UCA1 expression as compared to low expression

Two article about lncRNA UCA1 studied OS and were therefore included in meta-analysis [14, 15]. Both studies conducted multivariate Cox regression analysis and the data such as HR is therefore directly extracted and put into pooled analysis. We observed no heterogeneity between studies (I2 = 0%, *P* = 0.32) and therefore fixed effect model was applied. A combined HR of 1.49 (95% CI: 1.17–1.91, *P* = 0.001) for those patients with high expression of PVT1 was observed. We could then conclude that high expression of lncRNA UCA1 can be used as a predictor of poorer overall survival (Figure 7).

### MEG3

**Figure 8:**
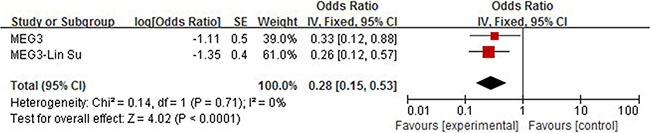
Forrest plots of studies evaluating hazard ratios of high MEG3 expression as compared to low expression

We carried out meta analysis with two articles describing the correlation between elevated expression of Meg3 and overall survival. The subsequent combined HR is shown in Figure 4. No heterogeneity among selected studies according to *Q*-test (chi2 = 0.14) and I-squared result (I2 = 0%, *P* = 071) was observed, so the fixed model was applied to calculate a pooled HR (HR = 0.28, 95% CI = 0.15–0.53, *P* < 0.0001), which indicated that elevated expression of MEG3 could positively affect patients' overall survival (Figure 8).

## DISCUSSION

The current meta-analysis investigating the correlation between lncRNAs and cancer prognosis, demonstrated that the over-expression of lncRNAs was an effective predictor of survival in a variety of cancers, in terms of both OS and EFS. For NSCLC, it is of great interest to identify its prognostic biomarkers, which can help cast light on the stratification of patients and make clinical decisions. In recent years, an increasing number of studies have proved the aberrant expression of lncRNAs in human cancer including NSCLC [49].

Our study included 36 recently published articles and a total number of 6267 patients, which is considered powerful enough to consolidate and perform the subgroup analyses. In this study, we listed 34 lncRNAs that were potential prognostic biomarkers for prognosis (Table 3). Our meta-analysis looked into six lncRNAs (PVT1, AFAP1-AS1, LINC01133 and ANRIL, UCA1, MALAT-1, MEG3) whose prognostic roles have been clearly demonstrated in two or more articles. The combined HRs suggested that elevated expressions of PVT1, AFAP1-AS1, LINC01133, ANRIL, UCA1, MALAT-1 and MEG3 were significantly correlated with patients' poor prognosis (Figures 2, 4, 5, 6, 7, 8). Although one study on ANRIL alone showed no statistical significance (HR = 2.23, 95% CI: 0.89–5.59, *P* = 0.09), the pooled outcome of two studies added convincing evidence that increased expression of ANRIL indicates shorter overall survival time (HR = 2.42, 95% CI: 1.40–4.19, *P* = 0.002). Due to the limitation of the study number, these conclusions need more clinical trials for verification. The heterogeneity of the population was probably due to the difference in source of population, the cut-off value of lncRNAs and the duration of follow-ups.

Distinct from earlier studies, this meta-analysis have summarized the prognostic role of all published lncRNAs in NSCLC and carried out pooled analysis on some certain lncRNAs with enough data. To the best of our knowledge, this is the first meta-analysis summarizing information about the prognostic value of all available lncRNAs in NSCLC patients. We strictly followed the literature inclusion criteria and all enrolled literatures were examined independently by two authors. Furthermore, we paid substantial attention to the details of study design and data reporting in quality assessment. We extracted data only of multivariate analysis to avoid the influence of heterogeneity among the included studies and to further explore the potential role of lncRNAs as prognostic biomarkers of NSCLC. As for Kaplan Meier survival curves, we carefully selected studies with valid information and strictly followed methods developed by Parmar, Williamson, and Tierney. Blurred curves were retouched with Microsoft Paint to make it precise for calculation. Furthermore, all data of extracted lncRNAs were based on frozen tissue samples of clear clinical origins. It was proven that the type of samples could influence the experimental outcomes in terms of RNASs detection [50]. All enrolled studies used qRT-PCR to measure lncRNAs which made pooled data from different studies more persuasive considering the consistent measurement background. Last but not least, all returned studies of our search strategy have been covered in this study which demonstrated the prognostic value of various lncRNA expression in NSCLC.

However, some details of our study need to be further refined. To start with, the number of eligible articles is relatively small, which lead to the relative insufficiency of studies in subgroup analyses. The possible cause for this was that studies reporting positive results were more likely to be published or that published literatures in other languages were missed during our search process. For the same reason, publication bias and sensitivity analyses were not performed, which might lead to the lack of statistical power. Second, the main ethnicities of the patients in our analysis were Asian. Thus, standardized analyses are expected in order to apply our results to other populations. Third, although all four sets of pooled outcomes of HR for OS in patients with high lncRNA expression were proven to be statistically significant (all HR > 2), some independent outcomes are not strong enough to have clinical value. Because empirically, a predictive HR value of more than 2.0 was considered to be statistically strong [51]. Although these results remain to be verified by larger numbers of clinical trials, they still possess statistic validity to reflex the general correlation of lncRNA expression with OS. The prognostic performance of lncRNAs in NSCLC has been proven. However, further clinical studies are warranted to Figure out the complicated molecular networks through which lncRNAs act to exert an influence on NSCLC patients.

## MATERIALS AND METHODS

### Search strategy

A comprehensive search was done via Pubmed database for literatures that analyzed the prognostic value of lncRNAs in NSCLC patients. Studies were selected using the varying combination of the following keywords: long non-coding RNAs, prognosis, lung cancer or NSCLC. The last search update was performed on May 19th , 2016. A second search was done on September 13th, 2016, using the following words: long non-coding RNAs, survival, lung cancer or NSCLC. Additional studies mentioned in those review articles were manually added to our evaluation list.

### Inclusion criteria

We referred to the guidelines of Preferred Reporting Items for Systematic Reviews and Meta-Analysis (PRISMA) Statement issued in 2009 as well as the checklist of the Dutch Cochrane Centre represented by MOOSE [52]. We then came up with a criteria for studies that are considered eligible for our full-text evaluation: (i) studies about the relation between lncRNAs expression in tumor or blood samples and prognosis of patients with NSCLC; (ii) the survival outcomes were measured with overall survival (OS) or event free survival (EFS) including disease free survival (DFS) and progression free survival ( PFS). The inclusion criteria is shown in Table 5.

**Table 5:** Summary of lncRNAs in the prognosis of NSCLC and authors' attitudes

Lnc RNA	Attitude	Sample size	HR provided	Reference
HOTAIR	Negative	119	yes	[15, 46]
PVT1	Negative	190	yes	[16, 17]
AFAP1-AS1	Negative	453	yes	[18, 19]
LINC01133	Negative	107	yes	[20, 21]
ANRIL	Negative	155	yes	[11, 22]
H19	Negative	70	yes	[29]
MALA T-1	Negative	180	yes	[32, 47]
Sox2ot	Negative	47	yes	[33]
UCA1	Negative	224	yes	[34, 36, 37]
MVIH	Negative	42	yes	[35]
CARLo-5	Negative	62	yes	[28]
LINC00473	Negative	469	yes	[48]
XIST	Negative	53	yes	[45]
NEAT1	Negative	96	yes	[44]
HNF1A-AS1	Negative	856	yes	[42]
LINC00342	Negative	1926	yes	[41]
RP11-21L23.2	Negative	196	No	[43]
GPR158-AS1	Negative	196	No	[43]
RP11-701P16.5	Negative	196	No	[43]
RP11-379F4.4	Negative	196	No	[43]
BC087858	−	38	yes	[40]
TUSC7	Positive	112	yes	[12]
HMlincRNA717	Positive	118	yes	[31]
CASC2	Positive	76	yes	[23]
PANDAR	Positive	140	yes	[24]
SPRY4-IT1	Positive	121	yes	[25]
TUG1	Positive	192	yes	[26]
BANCR	Positive	113	yes	[27]
GAS6-AS1	Positive	50	yes	[18, 19]
MEG3	Positive	62	yes	[38, 39]
CTD-2358C21.4	Positive	196	No	[43]
RP11-94L15.2	Positive	196	No	[43]
KCNK15-AS1	Positive	196	No	[43]
AC104134.2	Positive	196	No	[47]

Negative= Higher expression of the lncRNAs indicates poor prognosis.

Positive= Higher expression of the lncRNAs indicates better prognosis.

Studies were excluded based on any of the following conditions: (i) review articles, laboratory articles or letters; (ii) articles about the prognosis of other tumors or other markers. When two articles involving the same medical center with similar data, the article with a larger sample size was selected. Two authors independently selected studies, and disagreements were resolved by consulting a third author.

### Data extraction

All data were extracted independently by two authors and any disagreements were resolved by consensus and consultation with a third investigator. We extracted the results of multivariate Cox hazard regression analysis provided in the articles. However, if these data were not directly available, we extracted the log-rank *p value* and Kaplan–Meier survival curves of survival outcomes with the number of patients at risk in each expression group for further calculation. The following data were extracted: name of first author, investigated lncRNAs, number of patients, HR with 95% CI, *P value*, population, sample site, assay and survival outcome parameter.

### Statistical methods

All HRs and 95% confidence interval(CI) were calculated with Tierney's method. The logHR and SE (logHR) (SE) were recorded for aggregation of the survival outcomes of different long non-coding RNAs. Pooled analysis of the survival outcomes of specific lncRNAs was then performed. A test of heterogeneity of combined HRs was carried out using Cochran's *Q* test and Higgins I-squared statistic. *P value* of < 0.05 or I2 > 50% was considered statistically significant. A random effect model (Der Simonian and Laird method) was applied if heterogeneity was observed (*P* < 0.05 or I2 > 50%), otherwise the fixed effect model was used [53]. All *P* values were two sided and a *P value* of less than 0.05 was considered to be statistically significant.
